# Patient experiences with value-based healthcare interventions at the HIV outpatient clinic of the Erasmus Medical Centre

**DOI:** 10.1371/journal.pone.0304859

**Published:** 2024-06-03

**Authors:** Evelien S. van Hoorn, Nadine Y. Bassant, Hester F. Lingsma, Theodora E. de Vries-Sluijs

**Affiliations:** 1 Department of Public Health, Erasmus MC, University Medical Centre Rotterdam, Rotterdam, The Netherlands; 2 Department of Internal Medicine–Infectious Diseases, Erasmus MC, University Medical Centre Rotterdam, Rotterdam, The Netherlands; 3 Department of Medical Microbiology and Infectious Diseases, Erasmus MC, University Medical Centre Rotterdam, Rotterdam, The Netherlands; Kilimanjaro Clinical Research Institute, UNITED REPUBLIC OF TANZANIA

## Abstract

**Background:**

One of the aims of value-based healthcare (VBHC) is to deliver more patient-centred care. However, little is known about the effect of VBHC interventions on patient experiences. We aim to explore how patients experience VBHC as implemented in an HIV outpatient clinic in an academic hospital in the Netherlands.

**Methods:**

The HIV outpatient clinic of the Erasmus MC, Rotterdam, the Netherlands, an academic tertiary hospital, implemented a VBHC intervention consisting of 1) implementation of a generic quality of life questionnaire, administered before each visit, 2) a change in consultation schedule; from twice a year face-to-face to one face-to-face double consultation and one remote consultation per year, and 3) a change in consultation structure; from a single face-to-face consultation with the infectious diseases (ID) specialist to a double consultation in which the patient visits both the nurse and the ID specialist. Semi-structured interviews were held with Dutch or English-speaking adult patients, that had been a patient within Erasmus MC for more than 5 years, on their experiences with the implemented changes.

**Results:**

Thirty patients were interviewed. Patients had no objections towards completing the questionnaires especially if it could provide the professionals with additional information. Patients were primarily positive about the change in consultation schedule. For the yearly remote consultation they preferred a telephone-consultation above a video-consultation. The change in consultation structure ensured that more topics, including psychosocial and medical aspects could be discussed. Some patients did not see the added value of talking to two professionals on the same day or completing the quality of life questionnaire before their consultation.

**Conclusion:**

Patients are generally positive towards the VBHC interventions implemented at the HIV outpatient clinic. Our findings may inform further optimization of VBHC interventions and improve patient-centred care in outpatient HIV clinics.

## Introduction

The introduction of combination antiretroviral therapy (cART) has transformed HIV into a chronic, lifelong condition with a life expectancy comparable to the general population [1–4]. As a result, people living with HIV are aging and are at risk of developing treatment side effects in the long term (for example osteoporosis, renal function loss, lipodystrophy) and comorbidities such as hypertension, diabetes, cardiovascular disease, neuropathy and, liver and kidney disease [1, 3, 5]. Healthcare providers must pay increasing attention to the prevention and treatment of these comorbidities not traditionally associated with HIV. In addition, improving and ensuring a good health-related quality of life among people living with HIV has become more important [2, 4]. The transformation into a chronic disease has led to a change in the demand for care, corresponding procedures and the way care is delivered to people living with HIV.

One way to improve the care provision for people living with HIV is through the implementation of value-based healthcare (VBHC). In theory, VBHC enables healthcare systems to create more value for patients. Here patient value is the measurable improvement in a patient’s health outcome divided by the cost of achieving that improvement [6, 7]. Through the implementation of VBHC, healthcare systems are thought to improve patient experience and population health, reduce the per capita cost of healthcare, as well as improve healthcare professional experiences [6]. In the Netherlands, patient value is not so much perceived as an improvement in a person’s health outcome that come at a certain price, but rather as a result of the interactive patient-healthcare professional relationship which tends to the individual needs of each patient [8]. Using this definition of value, it is essential to improve the interaction between patient-healthcare professional during a consultation.

The Erasmus Medical Centre (Erasmus MC) in Rotterdam, the Netherlands started to implement VBHC with the implementation of a generic quality of life questionnaire to measure and monitor patient’s health outcomes. In addition, the HIV outpatient clinic tried to increase the value of the patient-healthcare professional interaction during a consultation, by making some changes to the schedule and structure of the consultations. This study aims to explore how patients experience the VBHC intervention as implemented at the HIV outpatient clinic of the Erasmus MC.

## Methods

### Study design

Semi-structured interviews were held to understand the patients perspective related to the implementation of a generic quality of life questionnaire and the changes in the structure and schedule of the consultations at the HIV outpatient clinic of the Erasmus MC, an academic tertiary hospital in the Netherlands.

The study was reviewed and determined to be exempt by Medical Ethical Research Committee of the Erasmus MC, University Medical Centre Rotterdam, Rotterdam, the Netherlands.

### Setting and intervention

The HIV outpatient clinic at the Erasmus MC, the Netherlands consists of a team of ten infectious diseases (ID) specialists, three nurse consultants, and one nurse practitioner. All ID specialists are responsible for the outpatient clinic for half a day and are supported by one nurse consultant/practitioner. Together the ID specialist and nurse consultant/practitioner provide care to approximately 2300 patients. The majority of these patients are male (76,4%) and approximately 50 percent are originally from the Netherlands. The care provision at the HIV outpatient clinic of the Erasmus MC was very diverse before the changes were implemented. The number of consultations per year and with whom the consultation took place, the ID specialist and/or nurse consultant/practitioner, could differ per patient.

The interventions implemented at the HIV outpatient clinic of the Erasmus MC consisted of three different elements. The first element, the implementation of the generic quality of life questionnaire is part of the wider Erasmus MC intervention to transition towards a VBHC way of working. This generic quality of life questionnaire consisted of the patient-reported outcomes measurement information system (PROMIS) Global Health which assesses an individual’s health on five domains (physical function, pain, fatigue, emotional distress, social health) as well as general health perception [9]. This questionnaire was supplemented with one additional question asking patients what they wish to discuss with the healthcare professional.

Subsequently, the HIV outpatient clinic changed the schedule and structure of their consultations. Firstly, instead of patients having to visit the outpatient clinic twice a year, a remote consultation was introduced to replace one of the face-to-face consultations. Secondly, to maximize the value of the patient visit to the outpatient clinic, the patient had a consultation with both the ID specialist and the nurse consultant/practitioner on the same day instead of visiting one of the two healthcare professionals. Preferably, the patient first visited the nurse consultant/practitioner immediately followed by a consultation with the ID specialist. Before this annual double consultation, patients are asked to complete the generic quality of life questionnaire as part of their standard care at the Erasmus MC.

### Participants and data collection

Patients were recruited for this study using a mixture of convenience and purpose sampling between April 5^th^ and June 21^st^ 2022. The nurse consultant/practitioner or ID specialist approached potential participants during a regularly scheduled visit at the HIV outpatient clinic asking them to participate in this study. Besides a verbal explanation of the study, potential participants received a patient information letter, which contained information about the goal and purpose of the study as well as information about anonymity and confidentiality. If the patient agreed to participate in the study, informed consent was signed and an appointment was made to conduct a telephone interview. This telephone interview occurred within one week after their visit to the HIV outpatient clinic to prevent recall bias. The telephone interview was expected to take approximately 15 minutes of the patients’ time.

Patients eligible for this study were 18 years of age or older, English or Dutch speaking, able to give informed consent, and have been a patient for more than 5 years at the HIV outpatient clinic of the Erasmus MC, the Netherlands. The latter inclusion criterion ensured that patients were able to compare their experiences with the care provided before and after the implemented changes at the HIV outpatient clinic. There were no requirements for eligibility involving the reason for their visit to the outpatient clinic or if they completed the generic quality of life questionnaire before their visit. Patients were recruited until data saturation was reached. Data saturation was reached when no new information was obtained from the interviews on all three VBHC interventions.

An interview protocol was developed by the research team based on the three main changes implemented at the HIV outpatient clinic. The interview guide included questions about the patients experience with 1) the generic quality of life questionnaire, 2) the changes in consultation schedule, and 3) the change in consultation structure. In addition, the interview guide contained prompt for potential follow-up questions that could be used to enrich the interview. The interview guide was developed specifically for this study and was not pilot tested before use. The complete interview guide can be found in the S1 File.

All interviews were conducted by three (one male, two female) medical students under the supervision of two researchers. The medical students were instructed on the qualitative methodology and had no prior knowledge of the patients’ medical history or of the interventions implemented at the HIV outpatient clinic. Each interview was conducted one-on-one, by telephone in a private setting. Before the start of the interview, the interviewer introduced themselves as a medical student, once more explained the goal and purpose of the study, asked permission for audio-recording and confirmed informed consent verbally.

### Data analysis

All interviews were audio-recorded and transcribed verbatim by the medical students. An initial codebook with pre-developed coding categories and subcategories was developed based on the research questions and topics covered in the interview guide. After the initial coding, the codebook was refined and expended with new codes that emerged during the coding process. Two researchers coded the transcripts in Microsoft Word and discussed potential discrepancies until consensus was reached. The interviews were analysed using a thematic analysis approach in which codes related to the same intervention were explored, combined and given meaning.

## Results

### Participants

Thirty patients participated in the study. All interviews were conducted between April 11^th^ and June 24^th^, 2022 and lasted between 7 to 23 minutes (mean 12 minutes). Most of the participants were male (73%) and originally from the Netherlands (73%). All participants visited an ID specialist and a nurse consultant/practitioner one week before their inclusion in the study, and 83% of the participants completed the generic quality of life questionnaire before their visit (Table 1).

**Table 1:** Participants’ demographics.

		Male (n = 22)	Female (n = 8)
**Mean age in years (range)**		51 (34–74)	48 (37–65)
**Country of origin**			
The Netherlands		18	4
Other		4	4
**Completed quality of life questionnaire**		20	5

### Patients are willing to complete the generic quality of life questionnaire

The first VBHC intervention consisted of the implementation of a quality of life questionnaire which patients were asked to fill in before their face-to-face consultation. The majority of the participants who completed the questionnaire responded positively to its implementation (n = 22, 88%). They expressed no objection to completing the questionnaire especially if it gives the specialist additional information about how they are feeling. To illustrate, one participant *(participant 8)* said, “Yes, fine. For me a small effort and if it works better for the specialist to have a starting point for the conversation then that’s fine. And if something is wrong, I will say it anyway. But it is fine to do every time.”

A small number of participants (n = 5, 17%) felt that the questionnaire did not provide any additional value, especially if the patients do not experience any health problems. As one participant *(participant 23)* put it, “Yes, I filled it in. However, it all feels very double. I filled it in and then at some point it was clear there are no issues, is there anything else? No. Yes, well, that questionnaire itself is annoying. I think such a questionnaire is a bureaucratic solution. I don’t find the system useful; I shall put it that way.”

Twenty-one participants were asked their opinion about the contents of the questionnaire. The majority of the participants (n = 19, 90%) thought that the content of the questionnaire was general but appropriate. Seven participants (33%) expressed the need for additional questions related to HIV and the use of medication, while thirteen participants (62%) did not see the added value of adding HIV related questions to the questionnaire. As one participant *(participant 17)* said, “I actually think that the combination of the lab results, this kind of questionnaires and the topics that come up during the conversations with the doctor and the nurse provide you with a good picture, a total picture, of how things are going. So I wouldn’t say I’m really missing things in the questionnaire because, for example, they wouldn’t come up otherwise.”

The five patients (17%) that did not complete the questionnaire provided several reasons why they did not complete the questionnaire. These reasons include not being able to access the questionnaire due to computer difficulties, uncertainty about the goal or purpose of the questionnaire, and a general perception that questionnaires are to tedious to complete. Patients also mentioned to be confused about the difference between the generic quality of life questionnaire and the COVID-19 questionnaire. The COVID-19 questionnaire was implemented during the COVID-19 pandemic to ask patients if they experienced COVID-19-related symptoms before their visit to the outpatient clinic. The reminder to complete the quality of life questionnaire was send during the same time period in which the patients received the COVID-19 questionnaire. As one participant *(participant 13)* illustrated, “No, I didn’t. No, I didn’t know either. I thought I was going to fill in the COVID questionnaire and later it turned out to be a completely different questionnaire.”

### Patients prefer a remote consultation instead of a face-to-face consultation

The second VBHC intervention consisted of a change in consultation schedule in which patients received an annual remote consultation instead of a second face-to-face consultation at the HIV outpatient clinic. When the participants were asked about this change, the majority (n = 22, 73%) responded positively and reported different advantages of a remote consultation including less travel and waiting time, not having to take time off from work and an increasing efficiency during the consultation. Several participants also argued that a remote consultation is acceptable and preferable if everything is going well and for discussing routine aspects of care like passing on lab results. As one interviewee *(participants 21)* put it, “I think that [one remote consultation and one face-to-face consultation] is perfect. That is totally the right balance. And about the telephone consult, yes less travel time. You do not have to go to the hospital. I just find that efficient. It will probably only be a short call with some data and updates. And the hospital visit will be a little longer. Yes, I really only see advantages”.

When asked about the participants past experiences with a remote consultation, 85% of the participants that answered this question (n = 27) reported to have experience with a telephone consult. A small number of participants (n = 4, 15%) also had experience with a video consult. When asked about their preferences for a telephone or video consult, almost all participants preferred a telephone consult above a video consult. The participants raised a number of issues related to video consults such as the need for a certain level of computer skills, availability of a webcam and the access to a private area to take the video call especially if the participant is at work. The participants that had experience with video consults, mentioned one advantage of video consults, namely the fixed time of the consult. In contrast to telephone consults where the specialist can call at any time during (a part of) the day, video consults are scheduled at a fixed time so the patient knows when to login into the system used for video consults. This fixed time of the consults was one of the reasons some participants preferred the video above a telephone consult.

### Patients see advantages in a double consultation with both the nurse specialist/consultant and ID specialist on the same day

The last VBHC intervention consisted of a change in consultation structure in which all patients visiting the HIV outpatient clinic have an annual face-to-face double consultation in which they first visit the nurse consultant/practitioner immediately followed by a consultation with the ID specialist. The majority of the participants (n = 24, 80%) reported to have followed this new consultation structure. Six participants (20%) reported to have first visited the ID specialist followed by a consultation with the nurse consultant/practitioner.

The majority of the participants (n = 18, 60%) commented that the addition of the consultation with the nurse consultant/practitioner positively influenced their care. They argued that the double consultation provided more structure to the outpatient clinic, led to a decrease in waiting time, and allowed for more attention to potential psychosocial problems that may or may not require further treatment. Some participants also felt that more topics were discussed and that there was more time to discuss new research initiatives in the field of HIV, their treatment and any questions they might have. As one interviewee *(participant 12)* put it, “I think it’s a very nice way of working because the nurse does the social department while the doctor focuses more on the medical business. The nurse asks about the home situation, how things are going at work and the social parts. Things like that you never discuss with your doctor”.

Four participants (13%) did not see the added value of visiting both the nurse consultant/practitioner and the ID specialist on the same day. They commented that during the early years of their HIV infection it was important to see both the nurse consultant/practitioner and ID specialist, while now that they are in the chronic phase without any medical problems seeing both healthcare professionals on the same day does not provide any additional value. They argue that the double consultation takes more time and that they have to repeat themselves to explain any potential problems to both the nurse consultant/practitioner and the ID specialist. For example, one interviewee *(participant 11)* said, “No, normally you discuss everything with one person and now you discuss a little with the doctor and a little with the nurse. … Because you see two people, I also think it [the consult] takes a little longer. Well, I prefer for my visit to the hospital to be as short as possible. I have been busy for a little over half an hour now. In the past it was only fifteen minutes. So that’s twice as long”. Moreover, these four participants indicated that they could find their way to the nurse consultant/practitioner if necessary.

## Discussion

The implementation of VBHC at the Erasmus MC incentivised the HIV outpatient clinic to increase the value of the patient-healthcare professional interaction during a consultation. To improve this interaction, the HIV outpatient clinic, subsequently to the implementation of the generic quality of life questionnaire, implemented two additional interventions related to the schedule and structure of the consultations. The consultations now consist of an annual face-to-face double consultation with both the ID specialist and nurse consultant/practitioner on the same day and one remote consultation per year. This study explored how patients experienced these changes.

We found that patients do not always see the added value of the generic quality of life questionnaire or the double consultation, especially if they do not experience any health problems or are in the chronic stage of their HIV infection. The majority of the patients however, responded positively towards both interventions. They expressed no objection towards completing the questionnaire if it provided the healthcare professional with additional information about how they are feeling. Patients also indicated that the addition of the consultation with the nurse consultant/practitioner to the consultation with the ID specialist allowed for more attention to potential psychosocial problems. This positive attitude towards more focus on the physical, mental and social situation of the patient is important since people living with HIV often experience anxiety, depression and stigma, which, among others, can lead to a decreased quality of life and treatment adherence [4, 10–17]. Overall, these findings confirm that informing the patient beforehand on the aim and relevance of an intervention, as well has how the intervention can contribute to better care provision is essential for its overall success [18–20].

With regards to the patient experiences with the change in consultation schedule, we found that patients prefer a telephone consultation instead of a face-to-face consultation if everything is going well or to discuss routine aspects of care. The acceptance of a remote consultation, the mentioned benefits of a remote consultation, the preference for a telephone consult and the barriers for a video consultation are consistent with findings of previous studies on the use of telehealth in HIV care [21, 22].

### Strengths and limitations

As far as we know, this is the first study focusing on patient experiences with the provision of care at an HIV outpatient clinic and provides a unique insight into patient experiences but also preferences with care provision.

This study, however, has several limitations. Firstly, the interviews were conducted by three medical students that had limited to no previous experiences in qualitative research and conducting interviews. This inexperience was noted during an interim analysis after the first few interviews in which the students did not follow the complete interview guide and neglected to ask follow-up questions for clarifications on the answers given by the patients. This resulted in variation in which questions were answered by the patients, the richness of the answers given by the patients and the need to include more patients to obtain data saturation. After the first few interviews, the students therefore received additional training on qualitative research and conducting interviews.

Secondly, the findings of this study might not be representative for all patient experiences at our HIV outpatient clinic because of potential selection bias. This study primarily included patients that completed the quality of life questionnaire before their face-to-face consultation at the HIV outpatient clinic. During the study period, only 39 percent of all the patients at the HIV outpatient clinic complete the generic quality of life questionnaire before their consultation. To ensure that all patients received the same level of care and attention, the domains of the quality of life questionnaire were discussed during the consultation regardless if the patient completed the generic quality of life questionnaire before their consultation.

To mitigate the potential influence of both the inexperience of the medical students and the selection bias, it was important to obtain data saturation. Data saturation was reached after 28 interviews when no new insights were gained in the patients experiences with the implemented changes at the HIV outpatient clinic. In this study, we specifically focused on obtaining data saturation in the experiences of both patients that completed the generic quality of life questionnaire and patients whom did not complete the questionnaire. This increased the generalizability of our results and ensured that the results of this study are representative of the current patient population of the HIV outpatient clinic.

Lastly, the care provision at the HIV outpatient clinic was diverse before the interventions were implemented. Some patients already received a separate consultation with the nurse consultant/practitioner and ID specialist every year before this annual double consultation was structurally implemented at the HIV outpatient clinic. In addition, because of the COVID-19 pandemic most patients gained experience with a remote consultation, since this was the only way in which care could be continued. During the pandemic remote consultations became an acceptable substitute for a face-to-face consultation [23, 24]. The diversity in care provision and use of remote consultations before the changes were implemented at the HIV outpatient clinic might have positively influenced patients experiences and their attitude towards the changes.

### Implications for further research and practice

This study shows that the implemented VBHC interventions were received positively by the patients of the HIV outpatient clinic of the Erasmus MC. This finding can be used to inform other healthcare professionals and organizations wishing to implement VBHC or to increase the value of the patient-healthcare professional interaction. Especially the patients’ positive attitude towards the implementation of the generic quality of life questionnaire is promising since this is often seen as essential to providing optimal individualised HIV care [4, 25, 26].

In this study patients mentioned that the fixed time of a video consultation was one of the reasons they preferred a video above a telephone consultation. It would be interesting to assess the possibility and implications of scheduling telephone consults with a fixed time on the workings of the HIV outpatient clinic and healthcare professional and patients experiences. Moreover, to further stimulate the dissemination of VBHC in this patient population both nationally as internationally, additional research could be performed to identify patient preference towards the addition of HIV specific questions to the generic quality of life questionnaire.

This study however also found that some patients do not see the added value of a quality of life questionnaire or a double consultation in which the patient visits both the ID specialist and the nurse consultant/practitioner on the same day, especially if they are in chronic phase of their HIV infection or do not experience any health problems. This might indicate that the perceived added value of the three interventions might be dependent on stage of the HIV infection and the current (psychosocial) health state of the patient. Additional research should be performed to further understand patients experiences and preferences with care provision, and to assess the possibility of care personalization in which the care provision is adapted to patients individual needs and preferences. More specifically, future research could focus on potential difference in patient experiences and preferences depending on patient subgroups, for example the older HIV patients, women of migrant background, young adults, and children transitioning from paediatric to adult care. Subsequently, future research could focus on improving and adapting the current VBHC interventions to match the needs of patients with low health literacy or with a language barrier to further stimulate care improvements for all patients with HIV.

## Conclusion

Patients with HIV primarily responded positively towards the VBHC interventions implemented at the HIV outpatient clinic. Patients are willing to complete a generic quality of life questionnaire before their consultation and see advantages in the addition of a consultation with the nurse consultant/practitioner to the consult with the ID specialist. The added value of these interventions, however, diminishes if patients do not experience any health problems or are in the chronic stage of their HIV infection. Moreover, a telephone consultation is an acceptable and preferable alternative to a face-to-face consultation, especially if patients do not experience any health problems and for discussing routine aspects of care. Additional research should be performed to understand patient experiences and preferences in care provision to further optimize the VBHC interventions and to improve patient-centred care in outpatient HIV clinics.

## Supporting information

S1 File Interview guide. Interview guide used by the medical students to collect patient experiences with the implemented changes at the HIV outpatient clinic.
(PDF)
