# Supplementary material for: Patient experiences with value-based healthcare interventions at the HIV outpatient clinic of the Erasmus Medical Centre
Source: PLoS One. 2024 Jun 3;19(6):e0304859. doi: 10.1371/journal.pone.0304859 (PMC11146710; doi:10.1371/journal.pone.0304859)
Supplement: S1 File — Interview guide used by the medical students to collect patient experiences with the implemented changes at the HIV outpatient clinic. (PDF) [file pone.0304859.s001.pdf]

Supporting information to:

Patient experiences with value-based healthcare interventions at the HIV outpatient clinic of the Erasmus Medical Centre

Evelien S. van Hoorn[1*], Nadine Y. Bassant[2], Hester F. Lingsma[1], Theodora E. de Vries-Sluijs[2,3]

[1] Department of Public Health, Erasmus MC, University Medical Centre Rotterdam, Rotterdam, The Netherlands

[2] Department of Internal Medicine – Infectious Diseases, Erasmus MC, University Medical Centre Rotterdam, Rotterdam, The Netherlands

[3] Department of Medical Microbiology and Infectious Diseases, Erasmus MC, University Medical Centre Rotterdam, Rotterdam, The Netherlands

* Corresponding author

E-mail: e.vanhoorn@erasmusmc.nl

Interview guide

Opening of the interview

Hello, this is <name interviewer> from the Erasmus MC. Am I speaking with <name participant>? I am < introduce yourself, mention your medical background and impartiality to this topic>. During your last visit to the HIV outpatient clinic at the Erasmus MC, you were asked if you would like to participate in a study to find out how patients experience the changes implemented at the HIV outpatient clinic. You indicated to your doctor or nurse consultant that you might want to participate in this study, is that still true? Do you want to participate in this study?

Thank you for your participation in this study. Is this a good time to start the interview? Before we start, may I record the interview?

**Interview data**	
Interviewer:	
Language interview:	
Date interview:	
**Information participant**	
Name participant:	
Gender participant:	
Age participant:	
**Interview**	
**Experience with the old situation at the outpatient clinic**	
Introduction text:<br>You have been a patient at the Erasmus MC for a number of years. As you have probably noticed, some changes have recently been implemented at the HIV outpatient clinic. These changes influence your visit to the outpatient clinic. We would like to hear from you what you think about the changes and how they influence your experiences with the outpatient clinic. Therefore, we would like to ask you some questions.<br><br>First, we would like to know how you experienced the care at the HIV outpatient clinic before the changes were implemented, i.e. how you experienced the old situation.	
Main questions	*Sub questions and prompts*

<table>
<tr><td>How did you experience the care provision at the HIV outpatient clinic before the changes were implemented?</td><td>- How many times did you visit the outpatient clinic during one year?
- How often did you have a consultation with the doctor? And how many times did you have a consultation with the nurse consultant or nurse specialist?
- How often did you contact your nurse consultant or nurse specialist?
- Was this enough for you?
- Was it clear to you when and for which questions you could contact your nurse?
- Did you miss anything during you visit to the outpatient clinic or in the contact with your doctor or nurse?</td></tr>
<tr><td colspan="2">Experience with the consultation structure of the outpatient clinic</td></tr>
<tr><td colspan="2">Introduction text:
This year we have implemented several changes at the HIV outpatient clinic. These changes influence how we provide care for patients with HIV, and your visit to the outpatient clinic proceeds somewhat differently than before. In addition, we have started offering telephone and video consultations.</td></tr>
<tr><td>Main questions</td><td>Sub questions and prompts</td></tr>
<tr><td>If you followed the new consultation structure at the HIV outpatient clinic, you first had a consultation with the nurse followed by a consultation with the doctor. What do you think of this new consultation structure?</td><td>- During your visit to the outpatient clinic, did you first have a consultation with the nurse followed by a consultation with the doctor? Or the other way around?
- How did you experience having a consultation with both the doctor and nurse on the same day?
- Do you feel that more topics are being discussed? Or that topics are explored more?
- What do you feel about the time the consultation and/or your visit to the outpatient clinic takes?
- What do you think about the frequency of consultations? Do you think once a year is too much or too little?
- Were you able to discuss everything that you wanted to with your doctor or nurse?
- Do you see advantages or disadvantages of the new consultation structure?</td></tr>
</table>

What do you think of the change to have one remote consultation instead of a 2nd consultation at the HIV outpatient clinic?	- Do you have previous experience with a telephone or video consultation?<br>- What are your experiences with a telephone or video consultation? Do you have a preference for the remote consultation?
**Questionnaire**	
Introduction text:<br>A few weeks before your consultation, you received an invitation for your consultation together with a letter. This letter contained information about the implementation of a generic quality of life questionnaire at the HIV outpatient clinic. Shortly before your consultation you received an e-mail with the request to complete this questionnaire.<br><br>We ask you to complete this questionnaire before your consultation at the HIV outpatient clinic because we would like to know how you are doing. Completing the questionnaire before your consultation might have a number of advantages for you, as well as for your doctor or nurse. For example, by completing the questionnaire you might be better prepared for your consultation. You can discuss what you want to ask your doctor or nurse with your relatives. The doctors and nurses can use the questionnaire to get an impression of how you are doing, and what you would like to discuss with them before your consultation. The overall aim of the implementation of the generic quality of life questionnaire is to improve your conversation with your doctor or nurse.	
Main questions	*Sub questions and prompts*
How did you experience completing the questionnaire before your consultation at the HIV outpatient clinic?	- Did you receive the information letter together with the invitation for your consultation? Did you read the letter?<br>- Was the letter understandable?<br>- Did the green paper draw your attention?<br>- Why did you (not) complete the questionnaire?<br>- What would help you to complete the questionnaire in the future?<br>- How much time did it take you to complete the questionnaire?<br>- Was it clear to you why we asked you to complete the questionnaire before your consultation?<br>- What did you think of the questions and topics?<br>- Did you miss any questions or topics?

<table>
<tr><td></td><td>
- What do you think of adding HIV related questions to this questionnaire?
- Would it help you or would you be willing the complete the questionnaire if more questions were directly related to HIV?
- At the end of the questionnaire it is possible to enter your own questions or topics you wish to ask or discuss with your doctor or nurse. Did you notice this? Did you use this opportunity?
</td></tr>
<tr><td colspan="2">Ideal situation from a patient perspective</td></tr>
<tr><td colspan="2">Introduction text:
Finally, we would like to know what your ideal visit to the HIV outpatient clinic would look like. Feel free to describe your ideal situation without any restrictions.</td></tr>
<tr><td>Main questions</td><td>Sub questions and prompts</td></tr>
<tr><td>What does your ideal visit to the Erasmus MC and the HIV outpatient clinic look like?</td><td>
- How often do you want to visit the hospital or outpatient clinic?
- Do you want to have a consultation with the doctor and/or nurse?
- How long should the consultation take?
- What kind of topics should be discussed?
- Is it necessary to do a blood withdrawal? And where/when should this blood withdrawal take place?
- Do you want to have the consultation in person, by telephone or video?
</td></tr>
</table>

Closing of the interview

This was the final question we wanted to ask you for this study. Do you have any questions for me? About the interview or the study? Do you want to be informed about the results of this study?

I will stop the recording. I want to thank for your time, effort and participation in this study.
